# Arbeit in der psychosozialen Versorgung von Kindern, Jugendlichen und Familien während der Covid-19-Pandemie – Ergebnisse einer qualitativen Interviewstudie in Wien und Niederösterreich

**DOI:** 10.1007/s11614-021-00463-y

**Published:** 2021-12-06

**Authors:** Andrea Jesser, Anna-Lena Mädge, Carina Maier, Jana Hierzer, Sylvia Dörfler, Martha Haslinger, Johanna Muckenhuber, Beate Schrank

**Affiliations:** 1 Karl Landsteiner Privatuniversität für Gesundheitswissenschaften, Krems an der Donau, Österreich; 2 Universität für Weiterbildung Krems (Donau-Universität Krems), Dr.-Karl-Dorrek-Straße 30, 3500 Krems, Österreich; 3 Forschungsgruppe D.O.T. – Die offene Tür – Ludwig Boltzmann Gesellschaft Wien, Karl Landsteiner Privatuniversität für Gesundheitswissenschaften, Krems, Österreich; 4 Institut für Soziologie, Goethe Universität Frankfurt am Main, Frankfurt am Main, Deutschland; 5 Österreichische Liga für Kinder und Jugendgesundheit, Wien, Österreich; 6 Technische Universität Wien, Wien, Österreich; 7 Institut für Soziale Arbeit, FH Joanneum – University of Applied Sciences, Graz, Österreich; 8 Abteilung für Erwachsenenpsychiatrie, Universitätsklinikum Tulln, Tulln, Österreich

**Keywords:** Psychosoziale Versorgung, Covid-19, Krise, Psychosocial care, Covid-19, Crisis

## Abstract

Die Vorgaben des „Social Distancing“ während der Corona-Pandemie stellten Angebote der psychosozialen Versorgung von Kindern, Jugendlichen und Familien, die traditionell auf persönlichem Kontakt beruhen, in der Durchführung vor neue Herausforderungen. In dieser qualitativen Studie wurde zu drei Erhebungszeitpunkten zwischen März und Oktober 2020 untersucht, wie sich die Pandemie speziell auf diese Angebote ausgewirkt hat und wie Mitarbeiter*innen und Führungspersonen psychosozialer Einrichtungen die Arbeit unter den veränderten Gegebenheiten erlebten. Ihre Arbeitssituation wird unter Zuhilfenahme der Konzepte des Arbeitskraftunternehmers und der Vulnerabilität untersucht. Es werden zwei Themenfelder vorgestellt, die sich in der Analyse der ersten beiden Erhebungszeiträume als zentral erwiesen haben: Das Spannungsfeld zwischen dem Schutz der eigenen Gesundheit und der Versorgungssicherheit für Klient*innen und das Belastungserleben von Praktiker*innen in Zusammenhang mit stark veränderten Arbeitsrealitäten in der Sozialen Arbeit und der eigenen Betroffenheit von der Corona-Pandemie. Deutlich wird das Bestreben der Fachkräfte, trotz schwieriger Rahmenbedingungen unterstützende Angebote während der Krise aufrechtzuerhalten. Es zeigen sich aber auch die Belastung und Überforderung der Praktiker*innen im Feld und es wird ersichtlich, dass adäquate Rahmenbedingungen sowohl zur Stärkung der psychosozialen Versorgung generell wie auch für die Bewältigung gesellschaftlicher Krisen notwendig sind.

## Abstract (en)

The requirements of “social distancing” during the Corona pandemic raised challenges for the implementation of psychosocial care services for children, adolescents, and families, which are traditionally reliant on face-to-face contact. Between March and October 2020, this qualitative study investigated the impact of the pandemic on the provision of psychosocial care at three points in time. We examined how staff and managers of psychosocial institutions experienced their work under the changing circumstances and what opportunities as well as risks they perceived for their work with clients. Their work situation is investigated by means of the concepts of the “Arbeitskraftunternehmer” and of vulnerability. This paper presents two topics that emerged from the analysis of the first two waves of data collection. The first expresses a conflict between practitioners need to protect their own health and their aspiration to maintain services. The second addresses practitioners’ experiences of stress due to their own affectedness by the crisis as well as significant changes in work realities. Our data show the strong effort of professionals to maintain supportive services during the crisis despite difficult conditions. However, it also shows the strain on social workers in the field and that adequate framework conditions are necessary both to strengthen psychosocial care in general and to cope with social crises on this scale.

## Einleitung

Dieser Beitrag nimmt Veränderungsprozesse in der Sozialen Arbeit mit Kindern, Jugendlichen und Familien in Österreich in den Blick. Die hier vorgestellte Studie fragt insbesondere danach, wie die Praktiker*innen selbst ihre Arbeit angesichts veränderter Gegebenheiten erleben. Im folgenden Kapitel wird zunächst ein kurzer Überblick über die Rahmenbedingungen psychosozialer Versorgung von Kindern, Jugendlichen und Familien in Österreich gegeben. Abschn. 1.2 geht auf die durch die Pandemie angestoßenen Transformationsprozesse und Herausforderungen im Feld der Sozialen Arbeit ein; daran anknüpfend wird in Abschn. 1.3 der bisherige internationale Forschungsstand zur Situation der Sozialen Arbeit während der Covid-19-Pandemie skizziert – Untersuchungen aus Österreich gibt es bislang keine. Im Weiteren werden in Abschn. 1.4 die Konzepte des Arbeitskraftunternehmers (Voß und Pongratz 1998) und der Vulnerabilität (Dahlvik und Reinprecht 2014) eingeführt, die herangezogen werden, um die Arbeitssituation der Praktiker*innen theoretisch einzuordnen sowie die mit der Pandemie einhergehenden Unsicherheiten zu untersuchen, mit denen die Beschäftigten konfrontiert sind. Nach einer Beschreibung des methodischen Vorgehens der Studie in Abschn. 2 werden in Abschn. 3 die wichtigsten Ergebnisse präsentiert und in Abschn. 4 unter Bezug auf die theoretischen Konzepte diskutiert.

### Psychosoziale Versorgung von Kindern Jugendlichen und Familien in Österreich

Die psychosoziale Versorgung von Kindern, Jugendlichen und Familien in Österreich ist interdisziplinär und sektorenübergreifend organisiert. Neben Angeboten der Sozialen Arbeit umfasst die psychosoziale Versorgung fachärztliche Leistungen sowie Angebote aus den Bereichen Psychologie und Psychotherapie. Sie erstreckt sich auf die Bereiche Gesundheit, Soziales, Kinder- und Jugendhilfe, Schule etc. (Bundesministerium für Soziales, Gesundheit, Pflege und Konsumentenschutz 2021). Der Schwerpunkt der Versorgung liegt auf extramuralen ambulanten Angeboten. Zusätzlich steht eine eingeschränkte Anzahl an Plätzen für die stationäre Aufnahme zur Verfügung.

Trotz starker Bemühungen in Richtung einer integrierten Versorgung ist die Versorgungslandschaft nach wie vor stark fragmentiert und entsprechend unübersichtlich (Kern und Sagerschnig 2017). Sie umfasst Träger aus öffentlicher und privater Hand, wodurch Angebote unterschiedlichen gesetzlichen Vorgaben und Förderstrukturen unterliegen sowie eine unterschiedliche strukturelle Ausrichtung zwischen ambulant und stationär aufweisen (Kern und Sagerschnig 2016). Die notwendige Koordination, Kommunikation und Kooperation, um diese Versorgungslandschaft effektiv und zielgerichtet zu gestalten, ist noch im Aufbau begriffen und gestaltet sich außerdem in jedem Bundesland anders (Friedl et al. 2020).

Angebote der psychosozialen Versorgung richten sich an Kinder, Jugendliche und Familien, die selbst Unterstützungsbedarf erleben, sowie an Familien, in denen eine Gefährdung des Kindeswohls angenommen wird. Der Zugang zu diesen Angeboten gestaltet sich aufgrund der Fragmentierung des Feldes teilweise schwierig. Es besteht eine hohe Dunkelziffer an Kindern und Jugendlichen, die aufgrund psychischer Erkrankungen Unterstützungsbedarf haben. Zudem reichen fachärztliche Leistungen nicht aus, um den Bedarf zu decken. Auch ambulante Angebote für Kinder und Jugendliche sind oft nicht ausreichend vorhanden.

### Transformation der Sozialen Arbeit in der Corona-Pandemie

Durch die Covid-19-Pandemie als globale Krise werden aktuell gesellschaftliche Problemlagen und Ungleichheiten besonders deutlich (Schmitt 2020). Vulnerabilitäten von Menschen in schwierigen Lebenssituationen verschärfen sich und soziale, ökonomische und politische Spaltungen in der Gesellschaft treten deutlicher als bisher zutage (Fisher et al. 2020). Das stellt Politik, Gesundheits‑, Bildungs- und soziale Sicherungssysteme vor große Herausforderungen (Seelmeyer und Zorn 2015) und auch die Soziale Arbeit als eigenständiges gesellschaftliches Teilsystem und als Profession ist gefordert, auf die stattfindenden gesellschaftlichen Transformationsprozesse zu reagieren.

Seit Ausbruch der Covid-19 Pandemie wurden mit dem Ziel der Senkung der Infektionszahlen Maßnahmen des Social Distancing – der physischen Distanzierung – gesetzt. In Österreich wurde im März 2020 ein erster Lockdown verhängt. Dies bedeutete unter anderem Ausgangsbeschränkungen, die Schließung von Gastronomie, Handel, Schulen und Kulturstätten, Betretungsverbote öffentlicher Räume und Besuchsverbote in Einrichtungen wie Krankenhäusern oder Pflegeheimen, das Verbot privater Zusammenkünfte und eine Maskenpflicht in öffentlichen Innenräumen. Diese Maßnahmen wurden zunächst schrittweise gelockert und ab 1. Mai 2020 gänzlich aufgehoben. Um steigende Infektionszahlen einzudämmen, wurden im September 2020 erneut eine Maskenpflicht und Beschränkungen in der Gastronomie und bei Kulturveranstaltungen eingeführt; ein zweiter und dritter Lockdown folgten Ende des Jahres.

Für die Angebote der psychosozialen Versorgung gab es in dieser Zeit keine gesonderten gesetzlichen Vorgaben. Das hatte eine enorme Handlungsunsicherheit zur Folge, sowohl innerhalb der Einrichtungen in Bezug auf die Aufrechterhaltung oder Einstellung von Angeboten als auch bei den einzelnen Fachkräften in der Gestaltung der direkten Kontakte mit KlientInnen. Dort, wo eine Orientierung an den allgemeinen gesetzlichen Maßnahmen stattfand, waren persönliche Begegnungen mit Klient*innen (z. B. bei Hausbesuchen) in ambulanten Settings nicht mehr möglich und offene, niederschwellige sowie anonyme Angebote im öffentlichen Raum konnten nur noch nach vorheriger Terminvereinbarung und Angabe von Adressdaten in Anspruch genommen werden. Dadurch kam es zu einem Wandel der bisher von Niederschwelligkeit geprägten Versorgungslandschaft zu plötzlich hochschwelligen Angeboten. Das traf insbesondere die Zielgruppe der Kinder, Jugendlichen und Eltern hart, deren Situation sich im Laufe der Pandemie zuspitzte. Spannungen in Familien nahmen zu und wurden sichtbar im Anstieg häuslicher Gewalt und infolge von Kriseninterventionen, Notunterbringungen und Inobhutnahmen (Schönig und Löwenstein 2020).

Die Pandemie führte weltweit zu Herausforderungen für die Soziale Arbeit. Um Angebote insbesondere für vulnerable Klient*innengruppen zu sichern, war experimentelles und kreatives Handeln erforderlich (Banks et al. 2020). Auf Basis einer umfassenden Digitalisierung kam es in vielen Ländern zu einer Adaption bzw. Neuentwicklung von Angeboten und mehr oder weniger „über Nacht“ wurden viele Kontakte mit Klient*innen auf Video, Chat oder Telefon umgestellt (Cook und Zschomler 2020). Auch in Österreich wurden digitale Angebote neu entwickelt, die vor der Pandemie kaum in diesen Settings angeboten wurden und für die es bis dahin weder eine Finanzierungsgrundlage noch technische und praktische Erfahrungen gab.

Während v. a. zu Beginn der Pandemie viele der ad-hoc implementierten Veränderungen im Feld der Sozialen Arbeit reaktiven Charakter hatten, plädieren international mittlerweile zahlreiche Autor*innen dafür, die Krise als Chance zu nutzen, gesellschaftliche Transformationsprozesse mitzugestalten (Nissen 2020; Pentini und Lorenz 2020; Truell und Crompton 2020). Als „Akteurin in gesellschaftlichen Veränderungen“ (Mayrhofer und Pilgram 2014) müsse sich die Soziale Arbeit dafür einsetzen, dass ihre Expertise um die reale Lage und die Bedürfnisse ihrer Zielgruppen in politischen Planungen berücksichtigt wird und sich in tragfähigen Arrangements, Strukturen und Handlungsstrategien niederschlägt. Diese seien nicht ausschließlich an ihrem ökonomischen Erfolg oder – wie es aktuell häufig passiert – an medizinischen Gesichtspunkten zu bemessen, sondern sollten „Fragen nach den sozialen Bedingungen menschlicher Würde und eines guten Lebens“ in den Fokus stellen (Scherr 2014). Andernfalls bringe sich die Soziale Arbeit in die Gefahr einer Deprofessionalisierung (Buschle und Meyer 2020; Wang et al. 2020).

### Arbeiten in Pandemiezeiten

Bisherigen Forschungen sowie Stellungnahmen der Fach- und Berufsverbände zufolge führte die Pandemie weltweit zu einer Verschärfung und (weiteren) Prekarisierung von Arbeitsbedingungen in vielen Tätigkeitsfeldern der Sozialen Arbeit sowie zu einer Reihe ethischer Dilemmata.

So kam es in Einrichtungen, die soziale Dienste einstellen oder reduzieren mussten, zu Kurzarbeit und Existenzgefährdungen, v. a. wenn Dienste über die Abrechnung von Einzelleistungen finanziert wurden, die nicht mehr erbracht werden konnten (Schönig und Löwenstein 2020). Parallel dazu sahen sich viele Einrichtungen mit einem zunehmenden Bedarf der von ihnen betreuten Zielgruppen und einer dadurch bedingten Intensivierung von Arbeit konfrontiert. Durch den Umstieg auf Telearbeit und den Wegfall von Wegstrecken in der mobilen Arbeit kam es darüber hinaus zu einer Arbeitsverdichtung sowie zusätzlichen Belastungen im Zusammenhang mit Betreuungspflichten und Homeschooling (Buschle und Meyer 2020). Die Abgrenzung zwischen Beruf und Privatleben war schwieriger aufrechtzuerhalten und musste von Praktiker*innen selbst vorgenommen werden (Cook et al. 2020). Weitere Konsequenzen der Umstellung auf Telearbeit waren fehlende Absprachen mit Vorgesetzten, der Wegfall von Fallbesprechungen sowie Möglichkeiten des kollegialen Austauschs (Buschle und Meyer 2020; Hardering 2021; Meyer und Buschle 2020). Teams geben die Gelegenheit, schwierige Situationen und Entscheidungen mit Kolleg*innen und Vorgesetzten zu besprechen und können helfen, mit Stress und emotionalen Belastungen besser umzugehen (Banks et al. 2020; Dominelli 2021). Zusammenarbeit und gegenseitige Unterstützung in Teams wurde durch die Arbeit im Homeoffice seltener und schwieriger, was für viele Praktiker*innen einen erheblichen Belastungsfaktor darstellte (Cook und Zschomler 2020) und aus Sicht der Praktiker*innen auch negative Auswirkungen auf die Qualität der eigenen Arbeit hatte (Meyer und Alsago 2021). Virtuelle Besprechungsformate konnten insofern keine Entlastung schaffen, als sie nicht über den informellen Charakter verfügten, der erforderlich gewesen wäre, um frei über Ängste, Ärger und Unsicherheiten in Bezug auf Fälle zu sprechen. Während kurze Gespräche im Büro zum Arbeitsalltag der Praktiker*innen gehörten, wurde die Entscheidung, Kolleg*innen im Homeoffice aktiv zu kontaktieren oft vermieden, um niemanden zusätzlich mit den eigenen Anliegen zu belasten (Cook et al. 2020).

Im Kontakt mit Klient*innen waren Praktiker*innen gezwungen, mit minimalen Ressourcen und mitunter ohne Richtlinien für die Praxis oder Unterstützung durch Vorgesetzte schwierige Entscheidungen zu treffen, u. a. darüber, welche Klient*innen Unterstützung bekommen konnten und für welche diese unter den gegebenen Umständen nicht möglich war (Banks et al. 2020; Truell und Crompton 2020). Zentral von Regierungen oder Einrichtungen vorgegebene Strategien zur Bekämpfung des Virus berücksichtigten die soziale Lage marginalisierter und vulnerabler Gruppen der Gesellschaft nur unzureichend (Wang et al. 2020) und schränkten deren Zugang zu notwendigen Angeboten der Unterstützung massiv ein (Banks et al. 2020). Zudem waren vielerorts die Bedingungen für die Umsetzung gesetzlicher Vorgaben nicht erfüllt. So war etwa die Einhaltung des angeordneten Mindestabstands, insbesondere in der Arbeit mit Kindern und Jugendlichen, nicht immer möglich und in vielen Fällen fehlte entsprechende Schutzausrüstung für die Arbeit in direktem Kontakt mit Klient*innen (Truell und Crompton 2020). Diese komplexen und problematischen Rahmenbedingungen führten dazu, dass Praktiker*innen im einzelnen Bedarfsfall selbst entscheiden mussten, ob gesetzliche Vorgaben und Richtlinien der Einrichtungen eingehalten oder diese auf Basis des eigenen professionellen Selbstverständnisses und berufsethischer Werte übertreten wurden – sofern es unter Abwägung der Risiken für alle Beteiligten als vertretbar erachtet wurde (Banks et al. 2020). Diese schwierige Situation löste bei Sozialarbeiter*innen intensive Gefühle aus: Angst um die eigene Gesundheit und die von anderen, Betroffenheit und Verzweiflung angesichts der Schicksale, mit denen sie ihre Tätigkeit tagtäglich konfrontierte, Ohnmacht und Hilflosigkeit, wenn sich Krisensituationen zuspitzten, und Scham darüber, im Zweifelsfall nicht mehr getan zu haben (Banks et al. 2020). Auch von Überlastung (Dominelli 2021; Meyer und Alsago 2021) und psychischen Folgen wie Schlaflosigkeit, Stress und Burnout wurde berichtet (Truell und Crompton 2020). Gerade in einer Situation, in der Fachkräfte ganz besonders auf ihre eigene physische und psychische Gesundheit achten sollten, wurde deutlich, dass sie über der Sorge um Klient*innen oftmals die Fürsorge für sich selbst in den Hintergrund stellten (Khan et al. 2020; Miller und Reddin Cassar 2021; Peinado und Anderson 2020).

Im folgenden Kapitel werden diese Befunde in Bezug zu den theoretischen Konzepten des Arbeitskraftunternehmers (Voß und Pongratz 1998) und der Vulnerabilität (Dahlvik und Reinprecht 2014) gebracht.

### Praktiker*innen als Unternehmer*innen ihrer eigenen Arbeitskraft

In der Sozialen Arbeit sind die Beschäftigten in den meisten Fällen angestellt tätig und damit ihren Vorgesetzten gegenüber weisungsgebunden. Dennoch ist ein wesentliches Kennzeichen ihrer Tätigkeit ein weitgehend inhaltlich eigenständiges Arbeiten mit ihren Klient*innen. Trotz häufiger Teilzeitanstellungen und geringer Bezahlung wird ein hohes Engagement erwartet und gehört ein hohes Engagement auch zum Selbstbild vieler Sozialarbeiter*innen. Zugleich erlebt die Soziale Arbeit eine anhaltende Ökonomisierung (Bakic et al. 2007; Mayrhofer und Pilgram 2014). Untersuchungen der Veränderungsprozesse innerhalb der Sozialen Arbeit deuten auf eine Entwicklung zu „wettbewerbsfähigen Dienstleistungsunternehmen“ (Kratzwald 2009) hin, die nicht nur formal stattfindet, sondern auch das Selbstverständnis der Beschäftigten prägen kann.

In der Soziologie wird die Kombination einer angestellten Beschäftigung mit vielen Merkmalen einer selbstständig-unternehmerischen Tätigkeit problematisiert, unter anderem mit den Konzepten des Arbeitskraftunternehmers und der Subjektivierung von Arbeit. Voß und Pongratz (1998) verstehen unter Arbeitskraftunternehmer*innen Beschäftigte, die einerseits in ihrer Tätigkeit über ein erweitertes Ausmaß an Selbstkontrolle verfügen, andererseits steht dieser Selbstkontrolle ein verstärkter ökonomischer Druck seitens der Arbeitgeber gegenüber: Beschäftigte müssen zunehmend mehr und schneller Leistung erbringen. An die Stelle direkter Anweisungen treten Anforderungen einer Leistungssteigerung im Zuge einer erweiterten Selbststeuerung und Selbstorganisation. Wesentlich ist dabei in der Konzeption von Voß und Pongratz, dass Arbeitskraftunternehmer*innen nicht mehr als Arbeitnehmer*innen denken, sondern in einem hohen Ausmaß die Anforderungen und Ziele der Arbeitgeber übernehmen. Im Feld der psychosozialen Versorgung scheint dies allgegenwärtig. Für viele Fachkräfte sind eine altruistische Grundhaltung und damit einhergehend das Bestreben, Menschen zu helfen, bereits zentrale Motivationen für die Berufswahl (Müller-Hermann 2012). Die Unternehmen, in den meisten Fällen Non-profit-Organisationen, verfolgen das gleiche Ziel der bestmöglichen Unterstützung von Klient*innen. In einem Kontext traditionell flacher Hierarchien treffen sich hier die Motivationen von Mitarbeiter*innen und ihrer Vorgesetzten und die zentralen Ziele der Einrichtungen. Doch was bedeutet das für die Arbeitnehmer*innen?

Die Handlungsspielräume in der konkreten Ausgestaltung der Tätigkeiten werden von vielen Fachkräften der Sozialen Arbeit geschätzt. Sie gehen jedoch häufig mit einem hohen Ausmaß an beruflichen Anforderungen einher und können sich auch negativ im Sinne von erhöhtem Stress und Burnout-Gefahr auf die Sozialarbeiter*innen auswirken (Bakic et al. 2007). Diese Dynamik wird befördert durch die Rahmenbedingungen ökonomisierter Sozialer Arbeit, wenn etwa keine ausreichenden zeitlichen, personellen etc. Ressourcen für die Ausübung der Arbeit zur Verfügung stehen oder unter Zeitdruck und auf der Grundlage von Effizienzvorgaben gearbeitet werden muss. Dahlvik und Reinprecht (2014) greifen in ihrem Beitrag über die psychosoziale Versorgung unter den Rahmenbedingungen zunehmender Ökonomisierung für die Analyse der Arbeitssituation der Sozialarbeiter*innen auf das Konzept der Vulnerabilität zurück. Sie arbeiten mit einem relationalen Verständnis von Vulnerabilität. Demnach sei Vulnerabilität kein personen- oder gruppenbezogenes Merkmal, sondern vielmehr ein Handlungskontext, in dem „[R]outinen außer Kraft gesetzt werden, Spielregeln an Klarheit verlieren und Normen an Kohärenz einbüßen“ (Dahlvik und Reinprecht 2014) und in dem Sozialarbeiter*innen in Interaktion mit ihren Klient*innen immer wieder aufs Neue versuchen müssen, ihre Handlungsfähigkeit zu erhalten. Dabei seien sie mit rechtlichen und organisationalen Rahmungen konfrontiert, die die Arbeit mit Klient*innen behindern, weil sie an realen Bedürfnissen und Problemlagen der Klient*innen vorbeisehen. Die komplexe Herausforderung der Sozialarbeiter*innen bestehe darin, in konkreten Interaktionssituationen mit KlientInnen „situationsspezifische Lösungen [zu] finden, die sowohl den Ansprüchen der Organisation als auch jenen der KlientInnen genügen, möglichst ohne dabei selbst auf der Strecke zu bleiben“ (Dahlvik und Reinprecht 2014). Die Autor*innen zeigen, wie einerseits kreative Lösungen entwickelt werden, andererseits der fortwährende Kampf um den Rückgewinn von Handlungssicherheit bei Sozialarbeiter*innen zu Überforderung führt. Sie problematisieren den Anspruch individueller Verantwortungsübernahme, der sich auch in den Befunden zur Arbeitssituation der Sozialarbeiter*innen während der Corona-Pandemie abzuzeichnen scheint. Durch die Pandemie und die erforderlichen Maßnahmen zu deren Eindämmung unterlagen Interaktionen mit Klient*innen einem permanenten Anpassungsdruck an sich verändernde Rahmenbedingungen mit einer sehr eingeschränkten Vorhersehbarkeit. Das hatte zur Folge, dass in der Praxis der Sozialen Arbeit immer wieder neue Figurationen entstanden und interaktiv gestaltet werden mussten. Dahlvik und Reinprecht beziehen sich in ihrem Beitrag auf Unsicherheiten, die in Zusammenhang mit der zunehmenden Ökonomisierung der Praxis der sozialen Dienste stehen. Mit dem relationalen Konzept von Vulnerabilität lassen sich aber auch die mit der Pandemie einhergehenden Ungewissheiten, Unsicherheiten und Risiken in den Blick nehmen, welche die tagtäglichen Interaktionen mit Klient*innen prägen, so etwa die Frage, ob und wie Schutzvorkehrungen und Abstandsregelungen eingehalten werden und dabei der Schutz der eigenen Person, der Schutz der Klient*innen und die Erfordernisse des professionellen Handelns zur Bewältigung der Situation abgewogen werden.

Bezugnehmend auf die These des Arbeitskraftunternehmers und das Konzept der Vulnerabilität sollen in diesem Artikel erste Ergebnisse der Studie „Wege durch die Corona-Krise von psychosozialen Unterstützungsangeboten für Kinder und Familien“ dargestellt werden. Die Studie untersuchte, wie Mitarbeiter*innen und Führungspersonen psychosozialer Einrichtungen für die Versorgung von Kindern, Jugendlichen und Familien ihre Arbeit seit Einführung des Covid-19-Maßnahmengesetzes in Österreich erleben und wie sich ihre Arbeit durch die Krise verändert hat. Dazu wurden in insgesamt drei Erhebungswellen qualitative Interviews geführt und inhaltsanalytisch ausgewertet. In diesem Artikel werden Ergebnisse der ersten beiden Erhebungswellen vorgestellt, die sich auf die Zeit bis inkl. Juli 2020 beziehen.

## Methodisches Vorgehen

Um Veränderungen in der psychosozialen Versorgung von Kindern, Jugendlichen und Familien zu erfassen, wurde ein Längsschnittstudiendesign entwickelt, in dem drei Erhebungszeitpunkte vorgesehen waren, zu denen ein Sample von insgesamt 30 Teilnehmer*innen wiederholt befragt werden sollte. Für die Rekrutierung der Teilnehmer*innen wurde auf die Netzwerke der Projektpartner zurückgegriffen, die v. a. in Wien und Niederösterreich tätig sind.^1^ Einbezogen wurden Mitarbeiter*innen aus Organisationen der psychosozialen Versorgung, wobei ambulante Beratungs- und Therapieangebote, Angebote der offenen Jugendarbeit und ambulanten Familienbegleitung sowie temporäre stationäre Angebote einbezogen wurden. Voraussetzung für eine Teilnahme war, dass das Angebot für die Familien kostenfrei war bzw. bei geringen finanziellen Ressourcen der Familien kostenfrei angeboten wurde sowie regulär auf direktem Kontakt beruhte. Dadurch sollte der Blick insbesondere auf Angebote gerichtet werden, die sich an besonders vulnerable gesellschaftliche Gruppen richten. Weiters wurde darauf geachtet, Unterstützungsangebote aus dem ländlichen und urbanen Raum zu berücksichtigen sowie Einrichtungen von unterschiedlicher Größe (Mitarbeiter*innen 2 bis ≥100) und Struktur (Vereine, GmbH etc.). Es wurden Fachkräfte aus Leitungsebenen sowie der direkten praktischen Arbeit in das Sample aufgenommen und bis zu zwei Teilnehmer*innen aus einer Einrichtung, sofern diese unterschiedliche Hierarchieebenen repräsentierten. Dadurch sollten unterschiedlichen Anforderungen sowie Wahrnehmungen der Veränderungen in den Blick gerückt werden. Von den 30 Teilnehmer*innen der Studie befanden sich zu den Erhebungszeitpunkten 19 Personen in einer Leitungsposition, 11 Personen waren als Mitarbeiter*innen tätig. Im Raum Wien waren 19 Befragte beschäftigt; 11 Personen hatten ihren Arbeitsplatz in Niederösterreich. Die Geschlechterverteilung des Samples lag bei 21 Frauen und 9 Männern. An der ersten Erhebung zwischen Mai und Juni 2020 nahmen alle 30 Personen teil, im zweiten Erhebungszeitraum im Juni und Juli 2020 sowie im dritten Erhebungszeitraum im September und Oktober 2020 nahmen jeweils 29 Personen an den Interviews teil.

Die Datenerhebung fand mittels semi-strukturierter Leitfadeninterviews (Willig 2008) statt. Folgende Themenschwerpunkte wurden behandelt: Arbeitsalltag, Kontakt mit Klient*innen, durch die Pandemie bedingte Veränderungen, Bewertung des derzeitigen Angebots sowie geplante Weiterentwicklung der Angebote. Die Interviews wurden angesichts der gesetzlichen Maßnahmen online über Videoplattformen geführt. Die Pandemie hat auch in der Forschung zu einem Digitalisierungsschub geführt und qualitative Datenerhebung über Online-Plattformen in die Diskussion gebracht (Lobe et al. 2020). Interviews online zu führen, wirft insbesondere forschungsethische Fragen in Bezug auf Datenschutz und Vertraulichkeit auf (Sugiura et al. 2017). Im Vorfeld der Erhebung wurden daher eine Recherche zu den Datenschutzvorgaben verschiedener Anbieter durchgeführt und eine Auswahl an Plattformen zusätzlich im Hinblick auf ihre Funktionalität (z. B. Tonqualität von Audioaufnahmen, Stabilität der Verbindung) getestet. Die Entscheidung viel zuletzt auf die Anbieter Zoom und BigBlueButton, wobei bei beiden Plattformen nur der Konferenzmodus für die Interviews genutzt wurde. Die Interviewpartner*innen erhielten bereits im Vorfeld des ersten Interviews die Studieninformation und Einwilligungserklärung. Aus methodologischer Sicht wird von Veränderungen der Interaktion und Kommunikation durch die Nutzung von Online-Plattformen ausgegangen (Turner 2002); Kontaktqualität, Gesprächsintensität und Informationsbereitschaft wurden jedoch durchgehend sehr positiv erlebt, was möglicherweise an der pandemie-bedingten, zunehmenden Vertrautheit mit Online-Kommunikation liegen könnte. Im Anschluss an die Interviews wurde das Datenmaterial transkribiert und mithilfe der Datenanalyse-Software NVivo ausgewertet. Der Interpretationsprozess verschränkte Grundprinzipien und Methoden der Grounded Theory (Glaser und Strauss 1998) mit inhaltsanalytischen Methoden (Mayring 2010). Nach den Prinzipien des theoretischen Samplings und der Zirkularität (Strauss und Corbin 1990) wurde eine Auswahl der bereits transkribierten Interviews parallel zur weiteren Datenerhebung offen codiert. Die entstandenen Konzepte wurden in wöchentlichen Analysesitzungen im Team diskutiert und schließlich zu Kategorien zusammengefasst. Nach Durcharbeitung des gesamten Materials wurden die Kategorien angelehnt an die inhaltlich strukturierende Inhaltsanalyse nach Mayring thematisch zusammengefasst und in einem Kategoriensystem zusammengestellt. Die Daten der zweiten Erhebungswelle wurden in weiterer Folge und in einem vergleichbaren Auswertungsprozess ebenfalls in das entwickelte Kategoriensystem eingearbeitet, wobei eine Erweiterung der Kategorien im Hinblick auf Veränderungen seit der ersten Welle vorgenommen wurde. Bereits die Auswertung der ersten Erhebungswelle ließ relevante Veränderungen der Arbeitskontexte erkennen, die sich in der Analyse der zweiten Erhebungswelle erhärteten. Die Auswertung der dritten Erhebungswelle konnte aus zeitlichen Gründen nicht in die Publikation einfließen.

## Ergebnisse der empirischen Untersuchung: Relevante Themenfelder in der psychosozialen Versorgung

Im Folgenden werden zwei Themenfelder dargestellt, die sich in der Auswertung der Interviews der ersten beiden Erhebungswellen als zentral erwiesen haben.

### Schutz der Gesundheit versus Versorgungssicherheit für Klient*innen

Die Frage nach dem Schutz der eigenen Gesundheit in der Pandemie wurde von vielen Interviewpartner*innen dem Bestreben gegenübergestellt, Versorgungssicherheit für Klient*innen herzustellen. Alle Befragten gaben an, während der Pandemie im Rahmen ihrer Möglichkeiten Unterstützung angeboten zu haben. Im direkten Kontakt mit Klient*innen wurden von vielen Interviewpartner*innen Abstriche in Bezug auf ihren Selbstschutz gemacht. Sie berichteten, das Ansteckungsrisiko bewusst in Kauf zu nehmen. Unter Bezugnahme auf die Betroffenheit und Bedürfnisse der Klient*innen wurde das Risiko für die eigene Person auch häufig heruntergespielt oder hintangestellt.

Und eigentlich war es bei den Familien so, dass […] wir eigentlich in keinerlei Schutz waren. Und […] irgendwann dann nach diesen 2 Wochen war ich dann auch in der Einstellung, das ist meine Arbeit, das ist meine Aufgabe, so sehe ich es und wenn ich krank werde, bin ich bei mir jetzt davon ausgegangen, es wird wie bei einer Grippe, es wird so ähnlich sein und dann liege ich halt 2 oder 3 Wochen im Bett und dann ist es auch durchgestanden. (Int_30_01)

Wichtig für das Verständnis der Situation ist, dass es keine Anforderung von Kostenträgern war, Angebote aufrechtzuerhalten. Dies hatte zur Folge, dass Einrichtungen mit der Verantwortung für die schwierige Entscheidung, ob Angebote fortgesetzt oder eingestellt werden sollten, alleine blieben. Wie diese Entscheidung innerhalb der Einrichtungen getroffen wurde war unterschiedlich. In einigen Fällen – insbesondere in größeren Organisationen – wurde sie von der Leitungsebene getroffen und es wurden entsprechende Richtlinien für die Durchführung von Angeboten erstellt. Erfolgte dieser Prozess direktiv, reagierten Mitarbeiter*innen häufig mit Widerstand und Ärger, v. a. wenn vor der Pandemie partizipative Entscheidungsfindungen üblich waren. Erfolgte der Prozess in Abstimmung zwischen Leitungsebene und Mitarbeiter*innen, erlebten Mitarbeiter*innen dies sehr positiv. In jedem Fall führten eindeutige Vorgaben innerhalb der Einrichtung dazu, dass Personen, die in direktem Kontakt mit Klient*innen standen, ihrer Verantwortung für die Aufrechterhaltung von Angeboten entbunden und dadurch entlastet wurden. Klare Entscheidungshierarchien und eine präsente Führung erlebten die interviewten Mitarbeiter*innen als unterstützend. Interviewpartner*innen berichteten aber auch davon, wie Vorgesetzte es umgingen, klare Vorgaben zu machen, zugleich aber die Verantwortung für Entscheidungen nicht explizit an ihre Mitarbeiter*innen übertrugen. Hier lag es in der Verantwortung der Mitarbeiter*innen, wie Angebote sowie Schutz- und Hygienemaßnahmen im Kontakt mit Klient*innen umgesetzt wurden, was als emotional besonders belastend benannt wurde.

Also ich hab […] gemerkt, wie wir so zwischen den Stühlen gesessen sind, 50 % vor Ort und 50 % im Home-Office im Telefonieren, dass mich das gestresst hat. Eben weil ich dann die Entscheidung treffen musste, welcher Familie erlaube ich zu kommen und welcher nicht. […] Man wusste nicht genau, es war keine hundertprozentige Ordnung so: gut das ist klar, sondern es ist uns überlassen worden. (Interview 29_02)

Neben der Frage der Verantwortlichkeit für Klient*innen erschwerten auch Unsicherheiten bezüglich der rechtlichen Situation die Entscheidungen der Interviewpartner*innen. In Ermangelung klarer gesetzlicher Vorgaben für die Angebote der psychosozialen Versorgung wurden die allgemeinen Sicherheitsmaßnahmen der Regierung für die Arbeit angewendet. Nahezu alle Interviewpartner*innen erlebten jedoch, dass manche Vorgaben in ihrem Arbeitsbereich nicht umgesetzt werden konnten, etwa das Tragen eines Mund-Nasen-Schutzes bei der psychotherapeutischen Arbeit mit Kindern. Interviewpartner*innen thematisierten die Sorge, dass eine Nicht-Einhaltung strafrechtliche Folgen nach sich ziehen könnte.

Trotz einer hohen Bereitschaft, sich dem gesundheitlichen Risiko auszusetzen, gab es Momente und Settings, in denen sich Interviewpartner*innen auf Grundlage einer Risikobewertung gegen eine Durchführung der Angebote entschieden. Es wurde von vielen Interviewpartner*innen als schwierig erlebt, Absagen oder Veränderungen in der Angebotsgestaltung gegenüber den Klient*innen zu thematisieren, insbesondere wenn dadurch Bedarfe nicht abgedeckt werden konnten.

Im Rahmen der zweiten Erhebung wurden gesundheitliche Risiken für die Praktiker*innen von allen Interviewpartner*innen deutlicher angesprochen, was darauf hinweist, dass die Wahrnehmung der Risiken anstieg. Während viele Befragte nach wie vor wenig Möglichkeiten sahen, das Dilemma Selbstschutz vs. Versorgungssicherheit befriedigend zu lösen und auf das Ende der Pandemie und die Rückkehr zu gewohnten Settings hofften, zeigte sich doch in der zweiten Erhebungswelle eine neue Selbstverständlichkeit im Umgang mit den Schutzmaßnahmen. Die Erfahrungen in der Krise hatten dazu genötigt, neue Wege zu gehen, um auf die eigene Sicherheit – sowie auf die Sicherheit der Klient*innen – zu achten. Es wurden mitunter Angebote wieder aufgegriffen, die während des ersten Lockdowns eingestellt oder auf Online-Settings umgestellt worden waren, weil die Arbeit mit Mund-Nasen-Schutz, Handschuhen, Trennscheibe etc. zur neuen Routine geworden war und Praktiker*innen wie Klient*innen gelernt hatten, fehlende mimische Reaktionen durch eine bewusstere Wahrnehmung des Augenkontakts und Blickes, der Körpersprache und Stimme zu kompensieren.

Im Selbstschutz schauen wir so gut wir können auf uns, aber mit Mund-Nasen-Schutz und Handschuhen würden wir wahrscheinlich in den Familien in der Betreuung drinnen bleiben und nicht so wie’s ganz am Anfang war. Beim ersten Lockdown war das alles auf Telefon und Fernkontakt, da hat man schnell gemerkt, dass das nicht funktioniert. (Int_30_02)

Andere beschrieben, wie die Notwendigkeit, vorhandene Angebote anzupassen, Chancen eröffnete, neue Online-Angebote durchzuführen und längerfristig in die Arbeit zu implementieren, die bis dahin aus zeitlichen und/oder finanziellen Gründen nicht umgesetzt werden konnten.

Neben der Gefährdung der eigenen Person thematisierten Interviewpartner*innen auch eine Gefährdung der Gesundheit der Klient*innen. Die Aufrechterhaltung von Angeboten konnte dazu beitragen, Klient*innen in Krisen zu stabilisieren und Situationen zu deeskalieren, gleichzeitig stellte der Kontakt mit Praktiker*innen für Klient*innen ein gesundheitliches Risiko dar.

Also es war dann schon auch die Argumentierbarkeit ja, dürfen wir überhaupt noch in die Familien hinein und ist das eigentlich, wenn das Jugendamt nicht mehr geht, mit welchem Argument gefährden wir eigentlich die Familien, wenn wir hineingehen oder schützen wir sie mehr. (Int_14_01).

Das war besonders dort der Fall, wenn es das Setting erschwerte oder sogar verhinderte, ausreichende Schutzmaßnahmen zu treffen. Beispielsweise erwies sich die Einhaltung eines Mindestabstandes bei Hausbesuchen in beengten Wohnverhältnissen als schwierig. Einige Interviewpartner*innen sprachen von negativen Auswirkungen auf die Beziehung zu Klient*innen, wenn diese sie plötzlich als Gefahr erlebten. Sie betonten, wie wichtig es für ihre Arbeit war, einen für alle Beteiligten passenden Umgang mit der Situation zu finden. Dafür bedurfte es einer sensiblen Kommunikation insbesondere über Schutzmaßnahmen und Sicherheitsvorkehrungen, Ängste und Befürchtungen bezüglich einer Ansteckung und deren Folgen für die Familien, Kinder und Jugendlichen.

### Arbeitsrealitäten in der Sozialen Arbeit während der Corona-Pandemie

Die Pandemie betrifft die gesamte Gesellschaft und hat damit eine bisher unbekannte Dynamik in die psychosoziale Unterstützung transportiert. Neben dem Bemühen, Klient*innen in der Krise zu begleiten, waren auch alle Befragten selbst von der Krise betroffen und spürten ihre Auswirkungen. Schwierig erlebten vor allem Interviewpartnerinnen mit Kindern die Situation. Sie schilderten Doppel- und Mehrfachbelastungen durch Betreuungspflichten und Homeschooling in Verbindung mit einer Berufstätigkeit im Homeoffice. Außerdem wurden in den Interviews wirtschaftliche Ängste thematisiert sowie Ängste um die eigene Gesundheit.

Und einige Mitarbeiterinnen natürlich sind auch abgetaucht, muss ich sagen. Und auch verständlicherweise abgetaucht, es hat ihnen Angst gemacht, sie haben selber auch wirtschaftliche Ängste gehabt, wie geht es weiter, so kann ich nicht arbeiten. Oder bin alleinerziehend, jetzt kommt überhaupt kein Geld mehr rein. (Int_13_01)

Insbesondere die umfassenden Veränderungen der Arbeitssituation in der psychosozialen Versorgung beschäftigten Interviewpartner*innen und wurden vielfach als belastend erlebt. Veränderungen wurden vor allem in Bezug auf die Rahmenbedingungen für die Arbeit und die Arbeitszeit geschildert.

- **Flexibler Umgang mit sich verändernden Rahmenbedingungen für die Arbeit**
  Die wiederholten Umstrukturierungen, die aus der Notwendigkeit resultierten, Angebote einzustellen bzw. anzupassen und neue Angebote zu entwickeln, erlebten viele Interviewpartner*innen als Herausforderung, die ihnen ein erhöhtes Maß an Flexibilität abforderte. Die häufige Veränderung und die Unklarheit der gesetzlichen Vorgaben wurden von den Praktiker*innen in diesem Zusammenhang als zusätzliche Belastung geschildert.
  Alle Interviewpartner*innen mussten in Zusammenhang mit der Anpassung von Angeboten neue und zusätzliche Aufgaben übernehmen. Für Personen in Leitungsfunktionen gehörten dazu darüber hinaus Verhandlungen mit Kostenträgern, die Beschaffung von Schutzausrüstung, die Erstellung von Informationsmaterialien, die Veränderung von Dienstplänen, die Kommunikation aktueller Vorgaben an Mitarbeiter*innen und vieles mehr.
  *„Ich habe dann nachher als Geschäftsführerin aktiv immer wieder in den Ministerien, weiß Gott wo angerufen, keine Antworten bekommen, verzögert Antworten gekriegt oder ganz unterschiedliche Antworten bis heute., […] also die einen haben gesagt, nein, wir wollen nicht, dass Ihre Mitarbeiter in Kurzarbeit gehen, die anderen haben gesagt, WAS Ihre Mitarbeiter sind noch nicht in Kurzarbeit? Die eine Juristin hat mir dann geschrieben, ja ich verstehe schon, dass Sie dort klare Anweisung haben wollen, aber Sie kriegen halt nur Empfehlungen und wie Sie die auslegen und welche Sie übernehmen, müssen Sie selbst entscheiden.“* (Int_21_01)
  Steigende Anforderungen an ihre Flexibilität empfanden Praktiker*innen auch in der Arbeit mit Klient*innen. Im direkten Kontakt mussten Schutz- und Hygiene-Maßnahmen eingehalten und umgesetzt werden, die die Kommunikation aufgrund fehlender Mimik und veränderter Stimme oftmals stark erschwerten. In vielen Fällen etablierten Einrichtungen Online-Angebote, die von Mitarbeiter*innen im Homeoffice durchgeführt werden konnten. Dies zog mehrere Schwierigkeiten nach sich. Zum einen vermischten sich der berufliche und der private Raum, was dort problematisch wurde, wo das Gefühl entstand, im Videokontakt mit Klient*innen die eigene Privatsphäre nicht ausreichend schützen zu können, etwa weil kein neutraler Hintergrund zur Verfügung stand und Klient*innen Einblicke in das eigene Zuhause bekamen. Zum anderen mangelte es Praktiker*innen und Klient*innen an Rückzugsräumen, was für die Durchführung der Angebote wie auch aus datenschutzrechtlicher Sicht schwierig sein konnte, beispielsweise wenn vertrauliche Gespräche mit Klient*Innen geführt werden mussten.
  *„Alleine aus Datenschutzgründen was wichtig ist, dass [man] dann nicht zu zweit irgendwo im Wohnzimmer herumsitzt oder wo auch immer. Also das ist schon eine ziemliche Herausforderung und ich habe dann persönlich schon auch geschaut, wo setze ich mich hin, weil ich muss die Familienbilder nicht im Hintergrund haben. Also schon sehr bewusst mir den Platz ausgesucht und dann gestaltet.“* (Int_15_01)
  Viele Interviewpartner*innen brachten zum Ausdruck, dass sie unter diesen Rahmenbedingungen nicht in der gewohnten Professionalität arbeiten konnten und diskutierten einen möglichen Qualitätsverlust der Angebote.
- **Ausdehnung der Arbeitszeit bei gleichzeitiger Umstellung auf Kurzarbeit**
  Es war ein zentrales Anliegen der Interviewpartner*innen, ihre Angebote trotz der herausfordernden Rahmenbedingungen für ihre Arbeit aufrechtzuerhalten und deren Qualität zu sichern. Möglich schien dies jedoch in den meisten Fällen nur durch einen verstärkten Arbeitseinsatz und die Bereitschaft zur Mehrarbeit.
  *„Das Ganze ist aber verbunden mit einem immens hohen Arbeitsaufwand gewesen. Wir haben alle miteinander etwa 30* *% unserer normalen Arbeitszeit noch einmal Überstunden gemacht.“ *(Int_12_01)
  Zugleich mussten viele Organisationen aufgrund von Vorgaben durch Kostenträger ihre Angestellten in Kurzarbeit schicken bzw. entschieden sich in einigen Fällen die Mitarbeiter*innen selbst für Kurzarbeit, um Ressourcen der Träger zu entlasten oder (Mehrfach‑)Belastungen (z. B. Zugehörigkeit zu einer Risikogruppe, familiäre Verpflichtungen) bewältigen zu können. Angesichts des erhöhten Bedarfs an Unterstützung seitens der Klient*innen, dessen Abdeckung eigentlich eine Ausweitung der Möglichkeiten für Überstunden erfordert hätte, bedeutete diese Maßnahme eine massive Arbeitsintensivierung.
  „Das war natürlich schwierig, weil alle meine Mitarbeiter*innen Teilzeit arbeiten und von der Teilzeit dann nochmal 50 % weggenommen worden sind. Das war eine sehr große Herausforderung und auch belastend, weil die Kolleg*innen gemerkt haben, es ist der Bedarf da, es steigt der Bedarf, aber wir haben nicht mehr, sondern weniger Ressourcen.“ (Int_06_01)
  Sie hatte zur Folge, dass Interviewpartner*innen ihre Arbeit in die freie Zeit verlagerten. Der tatsächliche Umfang der geleisteten Mehrarbeit scheint kaum dokumentiert worden zu sein, u. a. da Überstunden während der Kurzarbeit gesetzlich nicht erlaubt waren. In der zweiten Erhebung wurde deutlich, dass die Notwendigkeit, Mehrarbeit zu leisten, sich über einen längeren Zeitraum erstrecken konnte. Die Arbeitsanforderungen schienen nur bewältigbar, wenn die Arbeit auch in der Freizeit erledigt wurde und dies durchgängig über den gesamten Erhebungszeitraum hinweg. Dazu kam, dass die Umstellung der Angebote eine massive Veränderung der Arbeitszeiten nach sich ziehen konnte, besonders wenn aufgrund von Homeschooling Arbeitsstunden in die Abende oder Wochenenden verlegt wurden oder die Begleitung von akuten Krisen der Klient*innen eine erhöhte Erreichbarkeit erforderlich machte.

In Zusammenhang mit den beschriebenen Veränderungen der Arbeitssituation thematisierten mehrere Interviewpartner*innen ihre Sorge, mit der persönlichen Leistungs- oder Belastungsgrenze konfrontiert zu werden. In den Interviews des zweiten Erhebungszeitraumes kam es vielfach zur Nennung von Erschöpfungszuständen, auch von Personen, die sich in der ersten Erhebung selbst als wenig oder nicht belastet beschrieben hatten. Schwierige Situationen im Rahmen von Betreuungsbeziehungen konnten dadurch nicht mehr so gut bewältigt werden. Interviewpartner*innen gelang es schwerer, sich gegenüber den Geschehnissen im Arbeitskontext abzugrenzen und aufgrund der konstanten Mehrarbeit sowie familiärer Verpflichtungen war es kaum möglich, freie Zeit als Möglichkeit für Entspannung und Regeneration zu nutzen. Zusätzlich intensivierten sich in mehreren Arbeitsfeldern die Problemlagen der Klient*innen. Besonderes in der offenen Kinder- und Jugendarbeit wurde eine Zunahme an Aggressionen geschildert.

## Zusammenfassung und Diskussion

Die dargestellten Themenfelder geben Einblicke in den Arbeitsalltag in der psychosozialen Versorgung von Kindern, Jugendlichen und Familien während der Corona-Pandemie in Österreich und verdeutlichen die herausfordernde Situation der befragten Praktiker*innen, die auch schon in anderen Untersuchungen aufgezeigt wurde (Banks et al. 2020; Buschle und Meyer 2020; Dominelli 2021; Meyer und Buschle 2020; Schönig und Löwenstein 2020; Smessaert 2020; Truell und Crompton 2020). Sozialarbeiter*innen sahen sich mit einer allgemeinen gesellschaftlichen Verunsicherung konfrontiert, welche nicht nur die Situation der Klient*innen verschärfte, sondern sie auch selbst unmittelbar betraf (Richter und Zürcher 2020). Ein Fokus sozialer Arbeit und der Angebote in der psychosozialen Versorgung liegt im Abbau sozialer Unsicherheit, Ungleichheit und gesellschaftlicher Exklusion (Bakic et al. 2008; Kreckel 1997). Dieser Anspruch spiegelte sich auch in den Erzählungen der Interviewpartner*innen: so zeigte sich bei allen Befragten ein hohes Verantwortungsgefühl für ihre Klient*innen. Dieses gehört historisch zum Leitbild des Berufsfeldes und zum Selbstbild vieler Fachkräfte (Mührel 2003). Seit März arbeiteten die Praktiker*innen im Ausnahmezustand, um Klient*innen so viel Unterstützung wie möglich und notwendig zuteilwerden zu lassen. Durch neue Formen der Kontaktaufnahme und der Beziehungsarbeit sowie eine Adaption der Angebote fanden die Institutionen Wege durch die Krise und Möglichkeiten, trotz „Social Distancing“ das psychosoziale Versorgungsnetz weitgehend aufrechtzuerhalten.

Fehlende Vorgaben und Unklarheiten der gesetzlichen Regelungen für die psychosoziale Arbeit hatten dabei zur Folge, dass sich Praktiker*innen bei der Durchführung ihrer Arbeit immer wieder in Grauzonen bewegten. Das barg nicht nur gesundheitliche Risiken für die Praktiker*innen – denen u. a. mit Rationalisierungen und Verharmlosung begegnet wurde –, sondern erforderte vielfach eine erhöhte eigenverantwortliche Gestaltung der täglichen Arbeit, insbesondere im direkten Kontakt mit Klient*innen. In diesem Zusammenhang berichteten Interviewpartner*innen von der Herausforderung, zwischen den gesundheitlichen Risiken und dem Nutzen von Angeboten abzuwägen und zu entscheiden, welchen Klient*innen ein persönlicher Kontakt angeboten werden sollte – und welchen nicht. Dort, wo es in Einrichtungen vonseiten der Führungsebene keine konkreten Vorgaben für die Durchführung von Angeboten gab, lagen Entscheidungen allein bei den Praktiker*innen im Feld, die sich damit oftmals alleingelassen fühlten und angesichts der großen Unsicherheit und Risiken den Wunsch nach kooperativen Entscheidungen äußerten. Man kann ihre Situation mit einer Individualisierung von Verantwortung beschreiben, die zwar Freiheiten für die Praktiker*innen bringen konnte, aber auch Zwänge und Belastungen implizierte (Bakic et al. 2007). Für ein Verständnis der Situation eignet sich auch das Konzept der Vulnerabilität, wie es von Dahlvik und Reinprecht (2014) beschrieben wird. Durch die Pandemie sind Beschäftigte im Feld der Sozialen Arbeit mit einer in diesem Ausmaß neuen Ungewissheit konfrontiert, die bisherigen Sicherheiten den Boden entzieht. Institutionelle Abläufe und Routinen sowie rechtliche Regelungen verlieren ihre Gültigkeit. Neue Abläufe, z. B. digitale Settings, müssen erst implementiert werden und werfen fachliche, ethische und datenschutzrechtliche Fragen auf. Die zentral vorgegebenen rechtlichen Rahmenbedingungen berücksichtigen die Bedarfe der Zielgruppen der Sozialen Arbeit nicht ausreichend und können in der praktischen Arbeit nicht immer umgesetzt werden (z. B. Abstandsregeln). Um in diesem Kontext handlungsfähig zu bleiben, müssen Sozialarbeiter*innen fortwährend in den einzelnen Interaktionen mit Klient*innen situationsspezifisch Stabilität wiederherstellen. Im Versuch, eine Balance zwischen Interessen der Einrichtung, Interessen der Klient*innen und ihren eigenen Interessen zu finden, entwickeln sie individualisierte Strategien, um mit den neuen Situationen umzugehen. Dies spiegelt sich wider in den Erzählungen der Interviewpartner*innen über schwierige Entscheidungen bezüglich der Fragen, ob Klient*innenkontakte virtuell oder vor Ort stattfinden sollen, auf welche Art und Weise gesetzliche Maßnahmen umgesetzt werden, ob im direkten Kontakt Masken getragen werden, wie viel Abstand eingehalten wird etc. Auch unter regulären Bedingungen beinhaltet Soziale Arbeit regelmäßig eine Gratwanderung zwischen Involviertheit und Abgrenzung. Durch die Covid-19-Pandemie entstand jedoch die Notwendigkeit, die eigenen beruflichen Routinen neu zu entwerfen und sie den sich permanent verändernden Gefährdungslagen und deren Einschätzung durch Arbeitgeber*innen, Fachkräfte und Klient*innen anzupassen.

Auch die theoretischen Überlegungen der deutschen Arbeitssoziologie zur Subjektivierung von Arbeit eignen sich für die Erklärung der Situation der Sozialarbeiter*innen und rücken die gesellschaftliche Einbettung der Sozialen Arbeit in einen neoliberalen Arbeitskontext in den Fokus. Insbesondere die These vom „Arbeitskraftunternehmer“ (Voss und Pongratz 1998) erachten wir als zutreffend. Sie beschreibt u. a. die Ansprüche an Selbstregulierung und Selbstmanagement, mit denen sich ein neuer Typus von Arbeitskraft konfrontiert sieht. Praktiker*innen konnten – und mussten – ihre Arbeit selbstorganisiert gestalten. Ihre Erzählungen in den Interviews thematisierten vielfach Strategien des Selbstmanagements unter veränderten Arbeitsbedingungen durch Maßnahmen des „Social Distancing“, etwa dort, wo es um die Umsetzung von Schutz- und Hygienemaßnahmen ging, die Handhabung veränderter Kontaktzeiten mit Klient*innen oder die Durchführung von Maßnahmen aus dem Homeoffice. Die Erhöhung von Verantwortlichkeiten der Beschäftigten steht immer auch in einem Zusammenhang mit einer Flexibilisierung und Entgrenzung von Arbeit. Diese zeigten sich auf verschiedenen Ebenen im untersuchten Feld. So erhöhten Praktiker*innen mit einem großen Selbstverständnis ihr Arbeitspensum, um sich für Klient*innen einzusetzen. Das bedeutete in vielen Fällen eine Ausdehnung der Arbeit in die Freizeit bzw. eine Zunahme unbezahlter Arbeit in der Freizeit. Die Arbeit aus dem Homeoffice und der Verzicht auf die Nutzung von Büros oder Besprechungsräumen für Klient*innenkontakte stellten eine Lockerung bzw. Auflösung der räumlichen Bindung der Arbeit dar. Teilweise kam es zur Abnahme institutioneller Regulierungen (z. B. Vorgaben für die Gestaltung von Angeboten oder die Umsetzung von Schutz- und Hygiene-Maßnahmen). Und auch in Bezug auf die inhaltliche und organisatorische Ausgestaltung der Arbeit zeigten sich in den Daten Hinweise auf Flexibilisierungsprozesse (flexiblere Regelung von Arbeitsabläufen, wiederholte Adaption von Angeboten, kurzfristige Übernahme zusätzlicher und neuer Aufgaben etc.), die verdeutlichen, dass wir es in der psychosozialen Versorgung mit hochgradig individualisierten Beschäftigten zu tun haben. Der „Unternehmer“-Begriff verweist zudem auf die zunehmende Ökonomisierung von Arbeitskraft im Sinne eines unternehmerischen Umgangs mit der eigenen Arbeitskraft, der mit Gefahren von Selbstausbeutung und Scheitern verbunden ist, die für die Situation selbstständig Erwerbstätiger charakteristisch sind (Pongratz und Voß 2002). Dies zeigte sich bei den von uns interviewten Praktiker*innen v. a. dort, wo von freiwilliger und unbezahlter Mehrarbeit berichtet wurde, die notwendig war, um den (zum Teil selbst auferlegten) Ansprüchen an die Qualität der Versorgung von Klient*innen weiterhin gerecht zu werden. Vor dem Hintergrund der äußerst schwierigen Rahmenbedingungen für die Arbeit während der Pandemie und insbesondere auch der eigenen Betroffenheit von der Krise ist es wenig überraschend, dass Befragte von Überlastung und Erschöpfungszuständen berichteten und das insbesondere in der zweiten Erhebungswelle, als der Ausnahmezustand bereits über vier Monate andauerte.

Abschließend ist festzuhalten, dass die bereits in den vergangenen Jahren komplexer gewordenen Problemlagen der psychosozialen Arbeit mit Kindern und Jugendlichen (Schober und Wögerbauer 2020) um die mit der Pandemie verbundenen Herausforderungen erweitert bzw. durch diese verstärkt wurden (Meyer und Buschle 2020). Studien zu den Auswirkungen der Pandemie auf das subjektive Wohlbefinden zeigen eine Zunahme von Stressoren und das Wegfallen von Bewältigungsstrategien in der Allgemeinbevölkerung (Zacher und Rudolph 2021), wobei davon ausgegangen werden kann, dass die Auswirkungen ungleich verteilt sind und besonders jene von negativen Konsequenzen betroffen sind, die ohnehin benachteiligt sind (Österreichische Liga für Kinder- und Jugendgesundheit 2020a, b; Banks et al. 2020). So nahmen Praktiker*innen im Befragungszeitraum einen erhöhten Unterstützungsbedarf bei vielen Klient*innen wahr. Diesem zu begegnen und Angebote unter schwierigen Rahmenbedingungen aufrechtzuerhalten, brachte viele Praktiker*innen an ihre Belastungsgrenze.

## Fazit

Bei der Bekämpfung einer Pandemie kommt es auf die Einbindung vulnerabler Gruppen in die Gesellschaft an (Barrett 2007). Die Aufgabe der Akteur*innen in der psychosozialen Versorgung ist es, besonders diese Menschen zu unterstützen und zu stabilisieren. Doch fehlte diese Unterstützung schon vor der Pandemie einem Viertel aller Kinder in Österreich, wodurch die Pandemie wie ein Verstärker für bereits vorhandene gesellschaftliche Missstände wirkte (Österreichische Liga für Kinder- und Jugendgesundheit 2020a, b). Die diskutierten Aspekte verdeutlichen, dass die gegebene Krisensituation viele Fachkräfte vor plötzlich veränderte Arbeitssituationen und Herausforderungen stellte, auf die mit viel Flexibilität (zeitlich, örtlich, methodisch) und Einsatzbereitschaft reagiert wurde. Die vorliegende Studie bekräftigt bestehende Erkenntnisse darüber, dass die Corona-Pandemie und ihre Folgen zu einer Verschärfung der Arbeitsbedingungen und Veränderungen professionellen Handelns führte (Buschle und Meyer 2020; Meyer und Buschle 2020; Richter und Zürcher 2020). Fachkräfte in der psychosozialen Versorgung von Kindern, Jugendlichen und Familien übernahmen besonders viel (implizite sowie explizite) Verantwortung und nahmen sich dadurch vielen Herausforderungen dieser Krise an.

Im psychosozialen Arbeitsfeld sind jedoch nicht nur die Klient*innen, sondern auch die Fachkräfte selbst Akteur*innen in Handlungskontexten, die sich durch Unsicherheit, Ungeschütztheit und Ungewissheit charakterisieren. Diese Vulnerabilität der Fachkräfte wird während der Pandemie durch das Wechselspiel einer allgemeinen Betroffenheit durch Covid-19, wenig förderliche und oft unklare Rahmenbedingungen und eine Verunsicherung der eigenen Handlungspraxis verstärkt und in sozialen Interaktionen mit Klient*innen immer wieder aktualisiert. Individualisierte Strategien spielen für die Bewältigung der erlebten Unsicherheiten und Risiken eine große Rolle. Dieser Artikel plädiert dafür, dass sich Soziale Arbeit auf der Grundlage von professionellen Erfahrungen und berufsethischen Prinzipien dafür einsetzt, strukturelle Bedingungen zu schaffen, mithilfe derer der Individualisierung von Verantwortlichkeit entgegengewirkt werden kann. Die hohen persönlichen Kosten, die die Arbeit im Feld der psychosozialen Versorgung Praktiker*innen abfordern, müssen vermieden werden. Es gilt – unter Einbindung und Beteiligung der Akteur*innen – passende Rahmenbedingungen zu schaffen, die eine Handlungsfähigkeit und Planungssicherheit derjenigen sichern, die im Feld tätig sind. Dazu muss sich die Soziale Arbeit intensiver an politischen und öffentlichen Diskursen beteiligen (Wagner 2020) und ihre aktive Rolle in gesellschaftlichen Transformationsprozessen (Nissen 2020; Pentini und Lorenz 2020; Truell und Crompton 2020) ernst nehmen.

## Footnotes

1 Die Forschungsgruppe D.O.T. – Die offene Tür – setzt sich in ihrer Arbeit mit der Entwicklung von Unterstützungsangeboten für Kinder und Jugendliche in Wien und Niederösterreich zur Förderung sozialer Kompetenzen auseinander, mit dem Ziel, Chancengerechtigkeit zu fördern. In diesem Rahmen besteht seit 2019 eine Mitgliedschaft und enge Zusammenarbeit mit der Österreichischen Liga für Kinder- und Jugendgesundheit.

## References

[CR2] Bakic J, Diebäcker M, Hammer E (2008). Aktuelle Leitbegriffe der Sozialen Arbeit. Ein kritisches Handbuch.

[CR1] Bakic, Josef, Marc Diebäcker, und Elisabeth Hammer. 2007. Wiener Erklärung zur Ökonomisierung und Fachlichkeit in der Sozialen Arbeit. Positionspapier. http://www.armutskonferenz.at/files/bakic_ua_oekonomisierung_soziale_arbeit-2007.pdf. Zugegriffen: 2. Nov. 2021.

[CR3] Banks, Sarah, Tian Cai, Ed de Jonge, Jane Shears, Michelle Shum, Ana M. Sobočan, Kim Strom, Rory Truell, María Jesús Úriz und Merlinda Weinberg. 2020. Practising ethically during COVID-19: Social work challenges and responses. *International Social Work* 63(5):569–83. 10.1177/0020872820949614.

[CR4] Barrett S (2007). Why cooperate? The incentive to supply global public goods.

[CR5] Bundesministerium für Soziales, Gesundheit, Pflege und Konsumentenschutz. 2021. Psychosoziale Versorgung von Kindern und Jugendlichen. https://www.sozialministerium.at/Themen/Gesundheit/Gesundheitssystem/Gesundheitssystem-und-Qualitaetssicherung/Planung-und-spezielle-Versorgungsbereiche/Psychosoziale-Versorgung-von-Kindern-und-Jugendlichen.html. Zugegriffen: 30. Nov. 2021.

[CR6] Buschle C, Meyer N (2020). Soziale Arbeit im Ausnahmezustand?! Professionstheoretische Forschungsnotizen zur Corona-Pandemie. Soziale Passagen.

[CR7] Cook LL, Zschomler D (2020). Virtual home visits during the COVID-19 pandemic: social workers’ perspectives. Practice.

[CR8] Cook LL, Zschomler D, Biggart L, Carder S (2020). The team as a secure base revisited: remote working and resilience among child and family social workers during COVID-19. Journal of Children’s Services.

[CR9] Dahlvik J, Reinprecht C (2014). Zirkulation von Vulnerabilität. Wie in Hausbesuchen der Altenpflege soziale Unsicherheit erzeugt und bewältigt wird. Österreichische Zeitschrift für Soziologie.

[CR10] Dominelli L (2021). A green social work perspective on social work during the time of COVID-19. International Journal of Social Welfare.

[CR11] Fisher J, Languilaire J-C, Lawthom R, Nieuwenhuis R, Petts RJ, Runswick-Cole K, Yerkes MA (2020). Community, work, and family in times of COVID-19. Community, Work & Family.

[CR12] Friedl R, Ecker B, Karwautz A (2020). Kinder- und jugendpsychiatrische Versorgung 2019 in Österreich – Stufen der Versorgung, Ist-Stand und Ausblick. Neuropsychiatrie.

[CR13] Glaser B, Strauss A (1998). Grounded Theory. Strategien qualitativer Forschung.

[CR15] Hardering F (2021). Von der Arbeit 4.0 zum Sinn 4.0? Über das Sinnerleben in der Arbeit in Zeiten der Digitalisierung. ÖZS. Österreichische Zeitschrift für Soziologie.

[CR18] Kern D, Sagerschnig S (2016). Integrierte psychosoziale Versorgung von Kindern und Jugendlichen.

[CR19] Kern D, Sagerschnig S (2017). Integrierte psychosoziale Versorgung von Kindern und Jugendlichen. Abschlussbericht.

[CR20] Khan YH, Mallhi TH, Hadal Alotaibi N, Alzarea AI (2020). Work related stress factors among healthcare professionals during COVID-19 pandemic; a call for immediate action. Hospital Practice.

[CR22] Kratzwald B (2009). Innovativ, effizient, kundInnenorientiert. Soziale Trägerorganisationen als Dienstleistungsunternehmen. Soziales Kapital.

[CR23] Kreckel R (1997). Politische Soziologie der sozialen Ungleichheit.

[CR24] Lobe B, Morgan D, Hoffman KA (2020). Qualitative data collection in an era of social distancing. International Journal of Qualitative Methods.

[CR25] Mayrhofer H, Pilgram A (2014). Soziale Arbeit in gesellschaftlichen Transformationsprozessen. Österreichische Zeitschrift für Soziologie.

[CR26] Mayring P (2010). Qualitative Inhaltsanalyse. Grundlagen und Techniken.

[CR27] Meyer N, Alsago E (2021). Soziale Arbeit am Limit? Professionsbezogene Folgen veränderter Arbeitsbedingungen in der Corona Pandemie. Sozial Extra.

[CR28] Meyer, Nikolaus, und Christina Buschle. 2020. Soziale Arbeit in der Corona-Pandemie: Zwischen Überforderung und Marginalisierung. Empirische Trends und professionstheoretische Analysen zur Arbeitssituation im Lockdown. Working Paper 4/2020. *IUBH Discussion Papers *–* Sozialwissenschaften*. https://www.econstor.eu/handle/10419/222297. Zugegriffen: 2. Nov. 2021.

[CR29] Miller JJ, Reddin Cassar J (2021). Self-care among healthcare social workers: The impact of COVID-19. Social Work in Health Care.

[CR31] Mührel E (2003). Verantwortung: ein Ethos in der Sozialen Arbeit. *Sozialmagazin. Die Zeitschrift für*. Soziale Arbeit.

[CR32] Müller-Hermann S (2012). Berufswahl und Bewährung. Fallrekonstruktionen zu den Motivlagen von Studierenden der Sozialen Arbeit.

[CR34] Nissen L (2020). Social work and the future in a post-Covid 19 world: a foresight lens and a call to action for the profession. Journal of Technology in Human Services.

[CR35] Österreichische Liga für Kinder- und Jugendgesundheit (2020). Bericht zur Lage der Kinder- und Jugendgesundheit 2020. Kinder, Jugendliche und die Coronapandemie.

[CR36] Österreichische Liga für Kinder- und Jugendgesundheit. 2020b. Corona und die Kinder – Herausforderungen, Auswirkungen und Perspektiven. https://www.kinderjugendgesundheit.at/site/assets/files/1361/pressetext_pk_prasentation_kinderliga_lagebericht_18_11_2020.pdf. Zugegriffen: 2. Nov. 2021.

[CR37] Peinado M, Anderson KN (2020). Reducing social worker burnout during COVID-19. International Social Work.

[CR38] Pentini Aluffi A, Lorenz W (2020). The Corona crisis and the erosion of ‘the social’—giving a decisive voice to the social professions. European Journal of Social Work.

[CR39] Pongratz H-J, Voß G-G, Eichmann H, Kaupa I, Steiner K (2002). Unternehmer der eigenen Arbeitskraft. Reichweite und Folgen des Typus des Arbeitskraftunternehmers. Game Over? Neue Selbstständigkeit und New Economy nach dem Hype.

[CR41] Richter D, Zürcher S (2020). Psychiatrische Versorgung während der COVID-19-Pandemie. Psychiatrische Praxis.

[CR43] Scherr A (2014). Gesellschaftliche Krisen und ihre Folgen für die Soziale Arbeit. Österreichische Zeitschrift für Soziologie.

[CR44] Schmitt C (2020). COVID-19. Sozial extra.

[CR45] Schober, Christian, und Julia Wögerbauer. 2020. *Studie zur Entwicklung der Betreuungskomplexität von Kindern und Jugendlichen*. Wien: Kompetenzzentrum für Non-Profit-Organisationen und Social Entrepreneurship. https://www.wu.ac.at/fileadmin/wu/d/cc/npocompetence/09_NPO_Abgeschlossene_Projekte/NPO___SE_Kompetenzzentrum_Forschungsbericht_Entwicklung_der_Betreuungskomplexit%C3%A4t_von_Kindern_und_Jugendlichen_Langversion.pdf. Zugegriffen: 2. Nov. 2021.

[CR46] Schönig W, Löwenstein H (2020). Netzwerke Sozialer Arbeit im Corona-Krisenmodus: Folgen des Lockdowns und Perspektiven ihrer Systemrelevanz.

[CR42] Seelmeyer U, Zorn I (2015). Digitale Technologien in der Sozialen Arbeit – Zur Notwendigkeit einer technischen Reflexivität. Der pädagogische Blick.

[CR48] Smessaert, Angela. 2020. Wenn Kümmerer*innen selbst Hilfe brauchen… Die Auswirkungen der Corona-Krise auf die Kinder- und Jugendhilfe. Berlin: Arbeitsgemeinschaft für Kinder- und Jugendhilfe – AGJ. https://www.agj.de/fileadmin/files/positionen/2020/AGJ_Zwischenruf_Corona.pdf. Zugegriffen: 2. Nov. 2021.

[CR49] Strauss A, Corbin J (1990). Basics of qualitative research: grounded theory procedures and techniques.

[CR50] Sugiura L, Wiles R, Pope C (2017). Ethical challenges in online research: public/private perceptions. Research Ethics.

[CR51] Truell R, Crompton S (2020). To the top of the cliff How social work changed with COVID-19.

[CR52] Turner JH (2002). Face to face: toward a sociological theory of interpersonal behavior.

[CR54] Voß G-G, Pongratz H-J (1998). Der Arbeitskraftunternehmer: Eine Neue Grundform der Ware Arbeitskraft?. Kölner Zeitschrift für Soziologie und Sozialpsychologie.

[CR55] Wagner L (2020). Soziale Arbeit und „Corona“. Sozial extra.

[CR56] Wang Yeong-Tsyr K, Wen-Hui T, Tze-Yin C, Lee H-J (2020). Rethinking four social issues of the COVID-19 pandemic from social work perspectives. Asia Pacific Journal of Social Work and Development.

[CR58] Willig C (2008). Introducing qualitative research in psychology.

[CR59] Zacher H, Cort WR (2021). Individual differences and changes in subjective wellbeing during the early stages of the COVID-19 pandemic. American Psychologist.

